# Diffusion imaging markers of accelerated aging of the lower cingulum in subjective cognitive decline

**DOI:** 10.3389/fneur.2024.1360273

**Published:** 2024-05-09

**Authors:** Ryn Flaherty, Yu Veronica Sui, Arjun V. Masurkar, Rebecca A. Betensky, Henry Rusinek, Mariana Lazar

**Affiliations:** ^1^Center for Advanced Imaging Innovation and Research, Department of Radiology, New York University Grossman School of Medicine, New York, NY, United States; ^2^Vilcek Institute of Graduate Biomedical Sciences, New York University Grossman School of Medicine, New York, NY, United States; ^3^Department of Neurology, New York University Grossman School of Medicine, New York, NY, United States; ^4^Neuroscience Institute, New York University Grossman School of Medicine, New York, NY, United States; ^5^Department of Biostatistics, New York University School of Global Public Health, New York, NY, United States; ^6^Department of Psychiatry, New York University Grossman School of Medicine, New York, NY, United States

**Keywords:** Alzheimer’s disease, subjective cognitive decline, diffusion tensor imaging, magnetic resonance imaging, aging

## Abstract

**Introduction:**

Alzheimer’s Disease (AD) typically starts in the medial temporal lobe, then develops into a neurodegenerative cascade which spreads to other brain regions. People with subjective cognitive decline (SCD) are more likely to develop dementia, especially in the presence of amyloid pathology. Thus, we were interested in the white matter microstructure of the medial temporal lobe in SCD, specifically the lower cingulum bundle that leads into the hippocampus. Diffusion tensor imaging (DTI) has been shown to differentiate SCD participants who will progress to mild cognitive impairment from those who will not. However, the biology underlying these DTI metrics is unclear, and results in the medial temporal lobe have been inconsistent.

**Methods:**

To better characterize the microstructure of this region, we applied DTI to cognitively normal participants in the Cam-CAN database over the age of 55 with cognitive testing and diffusion MRI available (*N* = 325, 127 SCD). Diffusion MRI was processed to generate regional and voxel-wise diffusion tensor values in bilateral lower cingulum white matter, while T1-weighted MRI was processed to generate regional volume and cortical thickness in the medial temporal lobe white matter, entorhinal cortex, temporal pole, and hippocampus.

**Results:**

SCD participants had thinner cortex in bilateral entorhinal cortex and right temporal pole. No between-group differences were noted for any of the microstructural metrics of the lower cingulum. However, correlations with delayed story recall were significant for all diffusion microstructure metrics in the right lower cingulum in SCD, but not in controls, with a significant interaction effect. Additionally, the SCD group showed an accelerated aging effect in bilateral lower cingulum with MD, AxD, and RD.

**Discussion:**

The diffusion profiles observed in both interaction effects are suggestive of a mixed neuroinflammatory and neurodegenerative pathology. Left entorhinal cortical thinning correlated with decreased FA and increased RD, suggestive of demyelination. However, right entorhinal cortical thinning also correlated with increased AxD, suggestive of a mixed pathology. This may reflect combined pathologies implicated in early AD. DTI was more sensitive than cortical thickness to the associations between SCD, memory, and age. The combined effects of mixed pathology may increase the sensitivity of DTI metrics to variations with age and cognition.

## 1 Introduction

Early detection and treatment of Alzheimer’s Disease (AD) is paramount in the prevention of neuronal degeneration and dementia. AD and related dementias were the 7th leading cause of death worldwide in 2019, affecting 55.2 million people (1). As of 2021, disability adjusted life years caused by AD are expected to more than triple by 2050 if current trends continue, with a particularly high impact forecasted in low and middle income countries (2). AD is characterized by a long preclinical stage noted by the deposition of amyloid plaques and tau tangles, followed by a destructive cascade of neurodegeneration leading to mild cognitive impairment and progression to dementia (3, 4). This cascade typically begins in the medial temporal lobe (5), a region important for memory (6). Early screening provides the patient an opportunity for future planning and lifestyle interventions to delay onset (3, 7–9). Therapeutic intervention in the preclinic stage also may interrupt the disease process before the onset of the neurodegeneration cascade (10). As disease modifying medications that disrupt this cascade such as aducanumab and lecanemab (11–13) become commercially available, early detection becomes increasingly important.

A promising avenue for early detection of AD is the study of subjective cognitive decline (SCD). SCD describes patients who complain of reduced memory performance or other cognitive decline but score normally on cognitive testing (14, 15). In cognitively unimpaired individuals above the age of 60, the prevalence of SCD is around 25% (16). Both multicenter studies and meta-analyses show increased rates of conversion to dementia in participants with SCD (14, 17, 18), with one meta-analysis showing SCD patients were twice as likely to develop dementia (14). In cognitively normal patients with positive amyloid and tau biomarkers, those with SCD are five times more likely to progress to mild cognitive impairment or dementia than those without cognitive complaints (19). SCD is also a better predictor of amyloid pathology than standard cognitive measures (20).

Despite its promise as a risk factor for dementia, SCD remains poorly understood. As AD is associated with atrophy of gray matter structures in the temporal, parietal, and cingulate cortices (21–23), many previous studies in SCD have focused on characterizing volume and cortical thickness of these regions (24–30), with inconsistent results (31–33). Prior research has also examined microstructural alterations in SCD using diffusion tensor imaging (DTI). DTI provides several diffusivity metrics, including fractional anisotropy (FA), axial (AxD), radial (RD), and mean (MD) diffusivity, which are all differentially affected by pathology. In a 2–3 year longitudinal study, DTI was shown to differentiate SCD patients who will progress to mild cognitive impairment from those who will not (34). Patients that converted to mild cognitive impairment also showed reduced cortical thickness and volume in the medial temporal lobe (34). However, DTI results in the medial temporal lobe have been inconsistent (35–37), potentially due, at least in part, to the relatively small samples used by some of the previous studies.

Therefore, the primary goal of this study was to further examine microstructural white matter changes in the medial temporal lobe using DTI, including associations with age and memory function, in SCD and unaffected control participants from Cambridge Center for Ageing and Neuroscience (Cam-CAN) (38, 39). Cam-CAN offers a large database of high quality anatomical, diffusion, fMRI, and magnetization transfer images alongside detailed behavioral, cognitive, and demographic measures. One other study reported on the incidence of SCD in Cam-CAN (40), and another showed increased MD and reduced FA with age across the white matter (41). However, to our knowledge this is the first study to study the relationship of SCD to diffusion imaging, cortical thickness, or volumes for brain structures other than hippocampus in the Cam-CAN dataset.

We chose to focus on the lower cingulum, or the white matter bundle leading to the hippocampus, as a region of interest (ROI) because as part of the medial temporal lobe, it is one of the most common starting points for neurodegeneration and tau pathology in AD (4). It is also an important region for memory (42). Further, the axons of the lower cingulum project to nearby gray matter regions that are vulnerable to neurodegeneration early in AD, including the entorhinal cortex, parahippocampal cortex, and the hippocampus (43).

A secondary goal of this work was to test whether cingulum microstructural variations relate to brain atrophy. Changes in brain structure volume and cortical thickness in SCD versus controls were examined for the lower cingulum and several AD signature gray matter regions including the hippocampus, parahippocampal cortex, entorhinal cortex, and temporal pole. Macrostructural metrics in areas associated with significant group differences were used as proxies for atrophy to examine the association of cingulum microstructure with neurodegeneration.

Using the large number of high-quality scans of older adults with SCD available from Cam-CAN, we may be able to detect microstructural changes not normally visible in smaller cohorts, lending new insights into the biology underlying SCD.

## 2 Materials and methods

### 2.1 Participant interview and cognitive testing

This study utilized the Cam-CAN dataset from the University of Cambridge, available at https://cam-can.mrc-cbu.cam.ac.uk/ (38, 39). Participants underwent a comprehensive cognitive battery and medical history examination to screen for neurological or psychiatric disorders. More details on data collection can be found in (39). Of the 3,000 Cam-CAN participants (38), we focused on those over the age of 55 with both cognitive health and diffusion MRI data available for a final N of 325. Of these participants, 127 answered affirmatively to the question “Do you feel you have any problems with your memory?” These 127 participants were labeled as having subjective cognitive decline (SCD). The remaining 198 were used as control participants without SCD. Sex was determined by self-report. The Mini-Mental State Exam (MMSE) (44) was given as part of the home interview. Participants with an MMSE score lower than 24 or who failed to complete the MMSE were excluded (39). One control participant was missing MMSE scores in the dataset but was included in the analysis due to normal scores on all other tests and inclusion in the imaging cohort. Memory performance was assessed using story recall from WMS-III (45) during the home interview. Participants who passed screening had their memory tested in the clinic with an emotional memory (EM) task and a visual short-term memory (VSTM) task (39).

### 2.2 Magnetic resonance imaging acquisition and processing

The Cam-CAN MRI data was collected on a 3 T Siemens TIM Trio scanner with a 32-channel head coil. Diffusion weighted images included b values of 1,000 and 2000 s/mm^2^ with 30 gradient directions each (39). Three images with *b* = 0 were also collected for each participant. Image noise and Gibbs ringing were attenuated using MRtrix version 3 (RRID:SCR_006971) tools dwidenoise (46–48) and mrdegibbs (49, 50). Motion correction was achieved using FSL version 6.0.4 (RRID:SCR_002823) tool mcflirt (51). The FSL DTIFIT algorithm was used to fit the diffusion tensor model with standard linear regression to each voxel, as well as to generate FA, MD, RD, and AxD maps for each participant (52). All images then underwent nonlinear transforms to standard space using FSL’s Tract-Based Spatial Statistic (TBSS) pipeline (53). Rather than a skeleton mask, the lower right and left cingulum bundles were extracted from the ICBM-DTI-81 white-matter labels atlas (54) and employed as regions of interest (ROIs). The skeleton masks output by TBSS were not used in this analysis. Regional white matter volume of the cingulum bundle, cortical volume, and thickness of several grey matter regions were generated with FreeSurfer version 7 (RRID:SCR_001847) tools recon-all-clinical and synthseg (55), then visually quality controlled. Cortical structure ROIs analyzed were regions known to be susceptible to cortical thinning early in the Alzheimer’s Disease course and included parahippocampal, entorhinal and temporal pole cortical thickness, as well as hippocampus and lower cingulum volume (21, 23). Brain structure volumes were divided by the intracranial volume to account for individual differences in head size.

### 2.3 Statistical analysis

Differences between the two groups in age, memory performance, gender distribution, brain structure volume, cortical structure thickness, and lower cingulum diffusion microstructure were assessed with *t*-tests and chi-squared tests in R version 4.2 (RRID:SCR_000432). Outliers in the diffusion metric bilateral regional averages, cortical thicknesses, and volumes were presumed to be the result of image processing or acquisition errors and were excluded using Tukey’s method (56). Sensitivity analyses compared models evaluated with and without outliers. Averages of each measure for the left and right lower cingulum ROIs were generated in MATLAB R2023a (RRID:SCR_001622). We then fit the following linear regression models:


(1) $$\begin{array}{c} \mathrm{Metric}=\beta_{0}+\beta_{1}*\mathrm{storyRecall}+\beta_{2}*\mathrm{group}\\ +\beta_{3}*\mathrm{storyRecall}*\mathrm{group} \end{array}$$

(2) $$\mathrm{Metric}=\beta_{0}+\beta_{1}*\mathrm{age}+\beta_{2}*\mathrm{group}+\beta_{3}*\mathrm{age}*\mathrm{group}$$

(3) $$\begin{array}{c} \mathrm{Metric}=\beta_{0}+\beta_{1}*\mathrm{age}+\beta_{2}*\mathrm{storyRecall}\\ +\beta_{3}*\mathrm{age}*\mathrm{storyRecall} \end{array}$$

(4) $$\begin{array}{c} \mathrm{diffMetric}=\beta_{0}+\beta_{1}*\mathrm{atophyMetric}+\beta_{2}*\mathrm{group}\\ +\;\beta_{3}*\mathrm{atrophyMetric}*\mathrm{group} \end{array}$$

where *diffMetric* is the regional average of each metric from DTI, *atrophyMetric* is the regional average cortical thickness or brain structure volume, *Metric* includes both diffusion and atrophy metrics, *storyRecall* is performance on the delayed story recall task, and *group* denotes SCD status (SCD versus control). Models 1, 2, and 4 included the group as a variable. Model 3 was examined across groups. All four models were employed for each diffusion metric. As we were primarily interested in the diffusion metrics’ associations with atrophy, only cortical thicknesses and brain structure volumes that were different between groups (*p* < 0.05 and Cohen’s D > 0.25) were included in Model 4. The linear regression models were fit in R. *p*-values smaller than 0.05 were considered statistically significant. In addition, voxel-wise analyses with multiple comparisons correction of the diffusion metrics within the extent of the lower cingulum bundles were calculated with threshold-free cluster enhancement (TFCE) including family-wise error correction (57) using FSL’s randomise (58) with the same four models. While the voxel-wise analysis is more sensitive and allows for more robust multiple comparisons correction, linear regressions of regional averages allow for better visualization of effect sizes, slope directions, and interaction effects. Age and story recall were mean centered across groups for use with FSL’s randomise, as described in the FSL documentation.

The code used for image processing and statistical analysis is available at: https://github.com/rf2485/camcan_scd_dMRI_paper.

## 3 Results

### 3.1 Group differences between SCD and controls

128 SCD participants and 199 control participants had imaging available, for a total of 327. There were no statistically significant differences between groups in age or sex distribution (Table 1). Of the memory tests employed in this study, only story recall was statistically significant between groups (Table 2). SCD participants performed significantly worse than control participants on both immediate and delayed story recall. We only used the delayed story recall score in the rest of our statistical analyses as it had a larger Cohen’s *d* effect size (0.37) than Immediate Story Recall (0.33) and MMSE (0.13). Three SCD participants and two control participants were excluded from the cortical thickness and brain structure volume analyses due to segmentation errors, leaving 322 datasets available for further analyses. SCD participants had thinner cortex in bilateral entorhinal cortex and right temporal pole (Table 3). There were 3 outliers for left entorhinal cortex thickness, 2 outliers for right entorhinal cortex, and 5 outliers for the right temporal pole. The results were robust to the removal of outliers. There were no significant group differences in any other cortical thicknesses or brain structure volumes analyzed (Supplementary Tables S1, S2).

**Table 1:** Demographic characteristics of the control and subjective cognitive decline (SCD) groups.

	SCD (*N* = 128)	Control (*N* = 199)	Total (*N* = 327)	Test statistic	*p* value
*Sex*				*χ*^2^ = 0.238	0.625
FEMALE	64 (50.0%)	94 (47.2%)	158 (48.3%)		
MALE	64 (50.0%)	105 (52.8%)	169 (51.7%)		
*Age*				*t* = 1.04	0.299
Mean (SD)	71.481 (8.941)	70.424 (9.011)	70.837 (8.985)		
Range	55.250–87.670	55.170–88.920	55.170–88.920		
*Income*				*χ*^2^ = 1.26	0.939
Less than £18,000	29 (22.7%)	50 (25.1%)	79 (24.2%)		
£18,000 to 30,999	40 (31.2%)	55 (27.6%)	95 (29.1%)		
£31,000 to 51,999	29 (22.7%)	43 (21.6%)	72 (22.0%)		
£52,000 to 100,000	20 (15.6%)	31 (15.6%)	51 (15.6%)		
Greater than £100,000	5 (3.9%)	12 (6.0%)	17 (5.2%)		
Prefer not to answer	5 (3.9%)	8 (4.0%)	13 (4.0%)		
*Ethnicity*					
White	126 (98.4%)	194 (97.5%)	320 (97.9%)		
Mixed	0 (0.0%)	1 (0.5%)	1 (0.3%)		
Asian or Asian British	1 (0.8%)	2 (1.0%)	3 (0.9%)		
Black or Black British	0 (0.0%)	1 (0.5%)	1 (0.3%)		
Chinese	0 (0.0%)	0 (0.0%)	0 (0.0%)		
Other	0 (0.0%)	1 (0.5%)	1 (0.3%)		
Do not know	0 (0.0%)	0 (0.0%)	0 (0.0%)		
No answer	1 (0.8%)	0 (0.0%)	1 (0.3%)		
*Years of Education*				*t* = −0.571	0.568
Mean (SD)	19.410 (4.087)	19.697 (4.902)	19.584 (4.595)		
Range	14.000–33.000	2.000–52.000	2.000–52.000		

There were no between-group differences in sex, age, income, or years of education.

**Table 2:** Between-group comparison in memory performance.

	SCD	Control	Total	Test statistic	*p* value	Cohen’s D
MMSE				*t* = −1.12	0.264	−0.13
*N*	128	198 &	326			
Mean (SD)	28.547 (1.362)	28.717 (1.306)	28.650 (1.329)			
Range	25–30	25–30	25–30			
Immediate Story Recall**				*t* = −2.73	0.007	−0.33
*N*	128	199	327			
Mean (SD)	12.617 (4.542)	13.894 (3.381)	13.394 (3.920)			
Range	3–23	4–23	3–23			
Delayed Story Recall**				*t* = −3.20	0.002	−0.38
*N*	128	199	327			
Mean (SD)	10.461 (4.673)	12.040 (3.800)	11.422 (4.228)			
Range	0–22	0–21	0–22			

MMSE, Mini-Mental State Exam. **p < 0.01. & One control participant is missing MMSE scores. However, they scored normally on story recall and denied any memory problems. Thus, we opted to include them in the analysis.

**Table 3:** Mean cortical thickness for temporal regions of interest (in mm) for the control and subjective cognitive decline (SCD) groups.

	SCD (*N* = 125 &)	Control (*N* = 197 &)	Test statistic	*p* value	Cohen’s D
Left entorhinal *			*t* = −2.41	0.017	−0.27
Mean (SD)	2.947 (0.191)	3.001 (0.207)			
Range	2.544–3.400	2.463–3.493			
Right entorhinal **			*t* = −2.86	0.005	−0.32
Mean (SD)	3.060 (0.176)	3.119 (0.191)			
Range	2.620–3.484	2.606–3.605			
Left temporal pole			*t* = −0.527	0.599	−0.06
Mean (SD)	3.446 (0.178)	3.456 (0.161)			
Range	3.014–3.878	3.035–3.850			
Right temporal pole *			*t* = −2.23	0.026	−0.27
Mean (SD)	3.630 (0.197)	3.679 (0.174)			
Range	3.191–4.161	3.213–4.126			

Between-group comparisons indicate decreased cortical thickness in SCD in left and right entorhinal cortices and the right temporal pole. *p < 0.05 and **p < 0.01. & There were 327 participants over the age of 55 with anatomical T1w images available. Two control participants and three SCD participants were excluded from the cortical thickness and brain structure volume analyses due to segmentation errors, for a total of 322 segmentations as detailed in this table.

127 SCD participants and 198 control participants had diffusion weighted images available, for a total of 325. There were no significant group differences in any of the diffusion metrics examined (Table 4). The results were robust to the removal of outliers.

**Table 4:** Regional diffusion metric mean values for the subjective cognitive decline (SCD) and control groups in the left and right lower cingulum.

	SCD (*N* = 127)	Control (*N* = 198)	Test statistic	*p* value	Cohen’s D
Left FA			*t* = 0.217	0.829	−0.03
Mean (SD)	0.296 (0.032)	0.295 (0.035)			
Range	0.231–0.398	0.186–0.387			
Left MD			*t* = 1.13	0.259	0.07
Mean (SD)	0.621 (0.048)	0.625 (0.047)			
Range	0.491–0.738	0.482–0.744			
Left AxD			*t* = 0.680	0.497	0.09
Mean (SD)	0.821 (0.060)	0.820 (0.059)			
Range	0.646–0.945	0.648e–0.97			
Left RD			*t* = 0.974	0.331	0.06
Mean (SD)	0.521 (0.047)	0.523 (0.045)			
Range	0.414–0.637	0.389–0.647			
Right FA			*t* = 1.59	0.112	0.18
Mean (SD)	0.299 (0.037)	0.306 (0.036)			
Range	0.206–0.397	0.220–0.389			
Right MD			*t* = −0.647	0.518	−0.13
Mean (SD)	0.634 (0.051)	0.634 (0.046)			
Range	0.500–0.787	0.478e–0.730			
Right AxD			*t* = −0.304	0.761	−0.04
Mean (SD)	0.852 (0.060)	0.849 (0.058)			
Range	0.665–1.000	0.608–0.962			
Right RD			*t* = −1.28	0.201	−0.17
Mean (SD)	0.534 (0.051)	0.534 (0.046)			
Range	0.409–0.678	0. 407–0.633			

No significant between-group differences are noted for any of the metrics. Diffusivity values are reported with units of 10^−3^ mm^2^/s.

### 3.2 Correlations between imaging and memory performance

**Figure 1:**
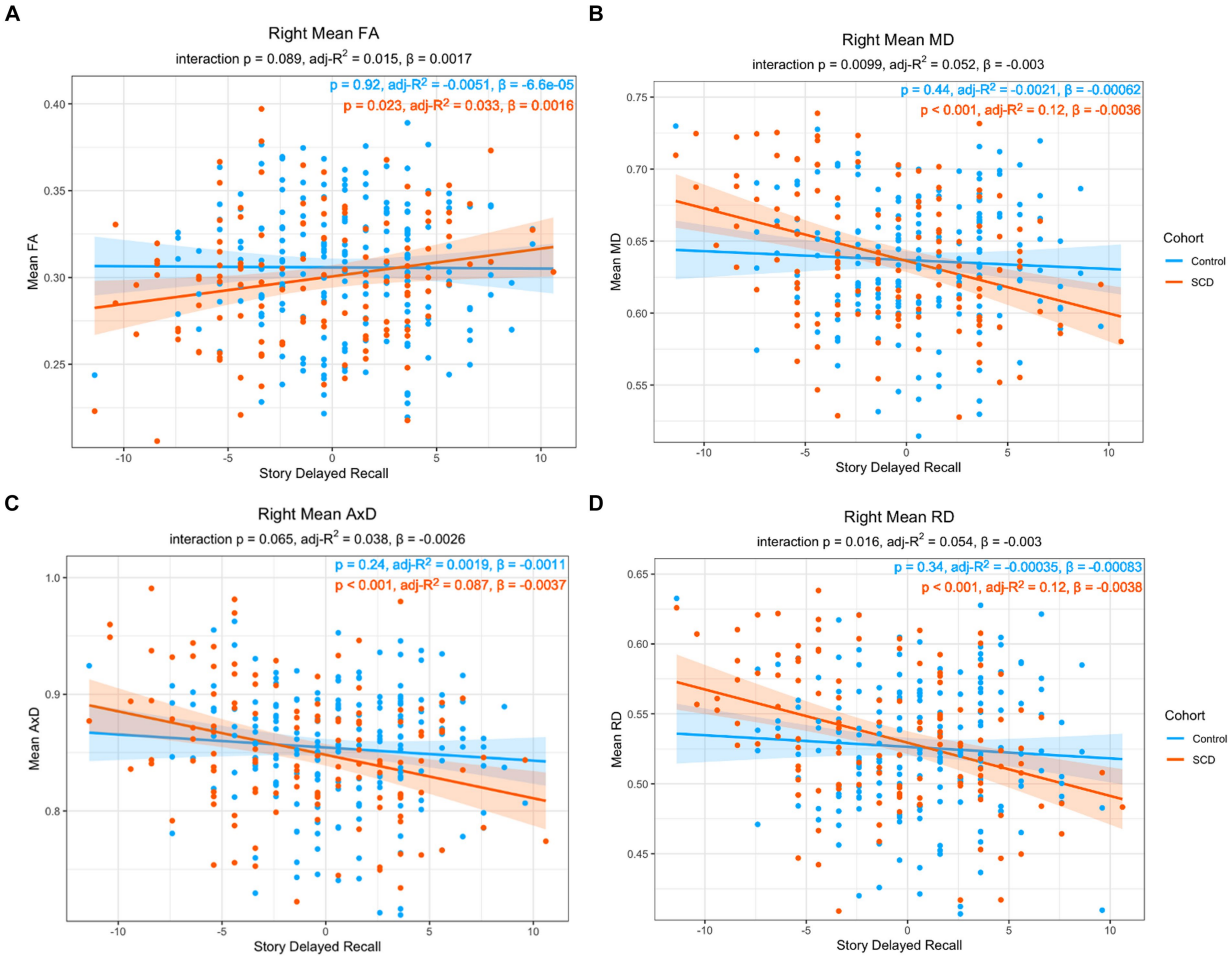
Linear regression of regional diffusion metric mean values in the whole right lower cingulum as a function of delayed story recall scores for (A) FA (B) MD (C) AxD and (D) RD. The p-values of the group by delayed story recall interaction are included in each chart subtitle. Interaction effects are significant for MD (B), and RD (D). The *p*-values of post-hoc within-group correlations between diffusion metrics and delayed story recall indicate a significant association between the diffusion metrics and memory function only in the SCD group in all diffusion metrics. Diffusivity values are reported with units of 10^−3^ mm^2^/s.

**Figure 2:**
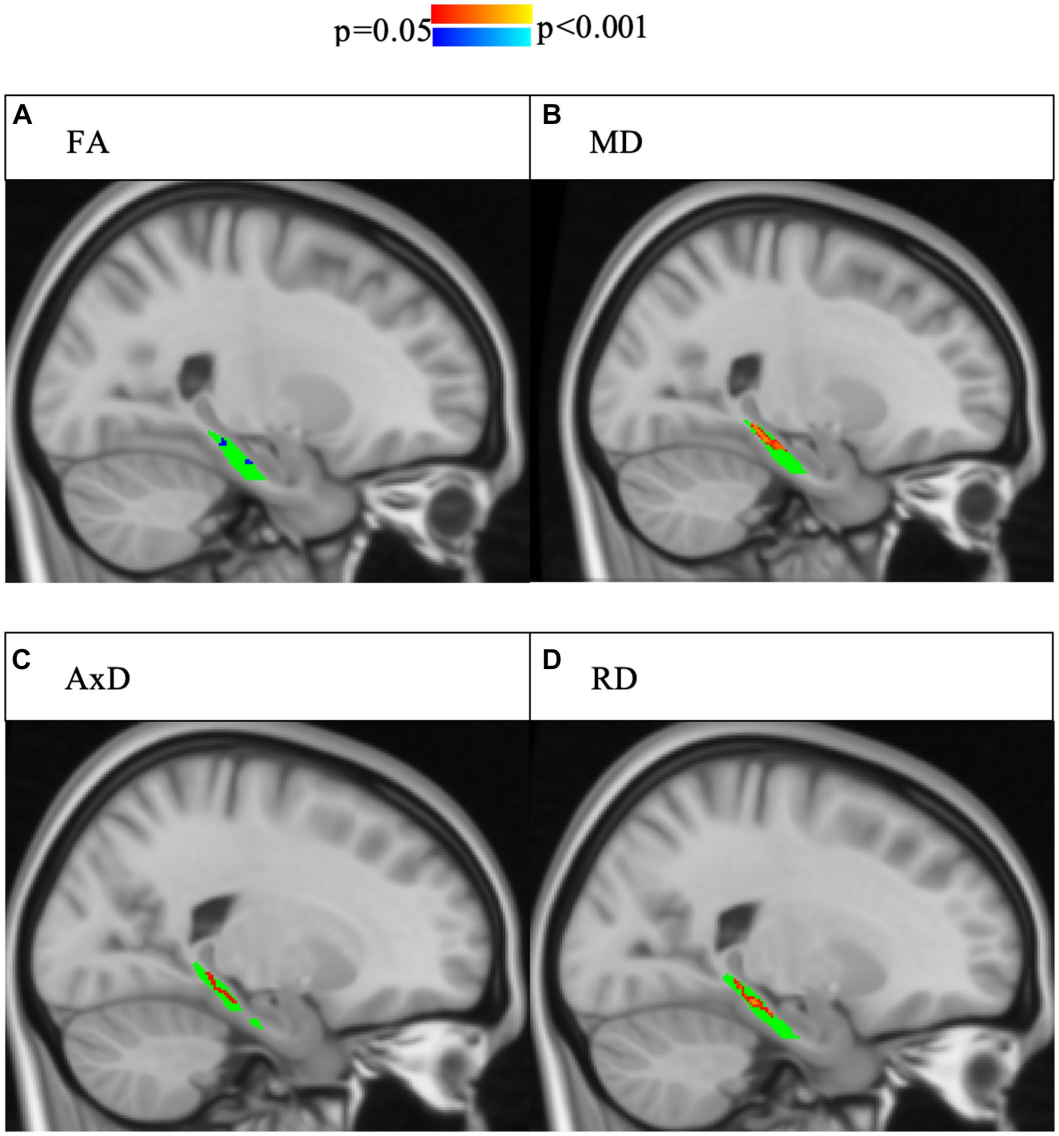
Interaction effects of cohort and delayed story recall score were confirmed by voxel-wise analysis with multiple comparisons correction of the whole right lower cingulum. Significant interactions (*p* < 0.05, corrected for multiple comparisons using threshold-free cluster enhancement) are highlighted, with blue indicating a more positive slope in SCD and red indicating a more negative slope in SCD. The region of interest (lower cingulum) is in green. Associations between increased memory scores and increased FA **(A)** as well as associations between decreased memory scores and increased **(B)** MD, **(C)** AD, and **(D)** RD were larger in SCD than controls.

For the right lower cingulum, there was a significant interaction effect between group and memory performance for regional mean MD, and RD (Model 1). Post-hoc analyses revealed that positive correlations with delayed story recall were significant for regional averages of FA, MD, AxD, and RD in SCD, but not in controls (Figure 1). Voxel-wise analyses with multiple comparisons correction confirmed this finding, with the addition of significant interaction effects for FA in a subset of voxels (Figure 2). There were no statistically significant findings between diffusion and memory in the left lower cingulum (Supplementary Table S3), nor were there any significant findings between memory and cortical thickness of grey matter regions studied (Supplementary Table S4). All findings were robust to the removal of outliers.

### 3.3 Correlations between imaging and age

**Figure 3:**
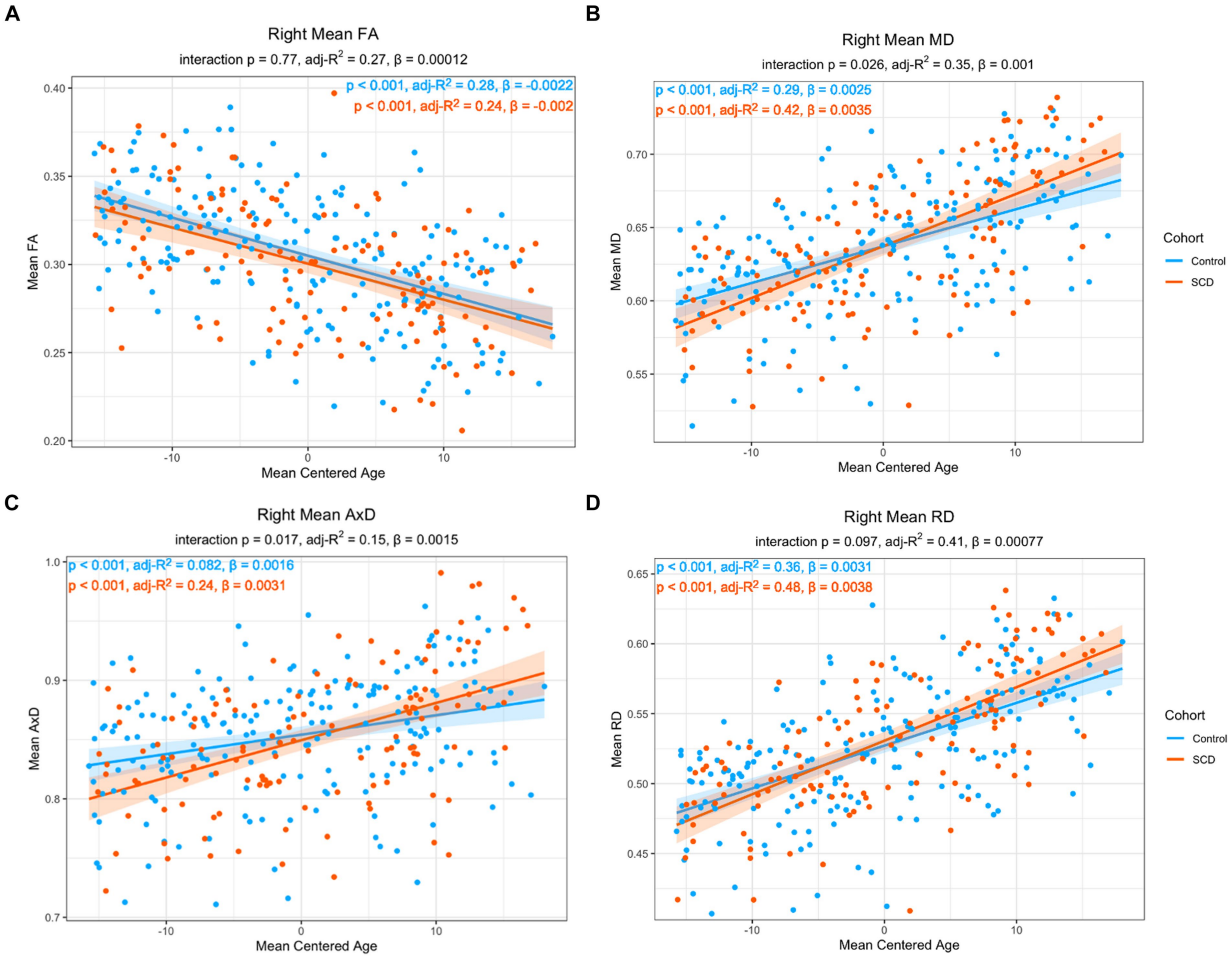
Linear regression of regional diffusion metric mean values in the whole right lower cingulum as a function of age for (A) FA (B) MD (C) AxD and (D) RD. The *p*-values of the group by age interaction are included in each chart subtitle. The interaction effects are significant for MD (B) and AxD (C) The adjusted R2 of post-hoc within-group correlations between diffusion metrics and age indicate a stronger association between the diffusion metrics and age in the SCD group in MD (B), AxD (C), and RD (D). Diffusivity values are reported with units of 10^−3^ mm^2^/s.

**Figure 4:**
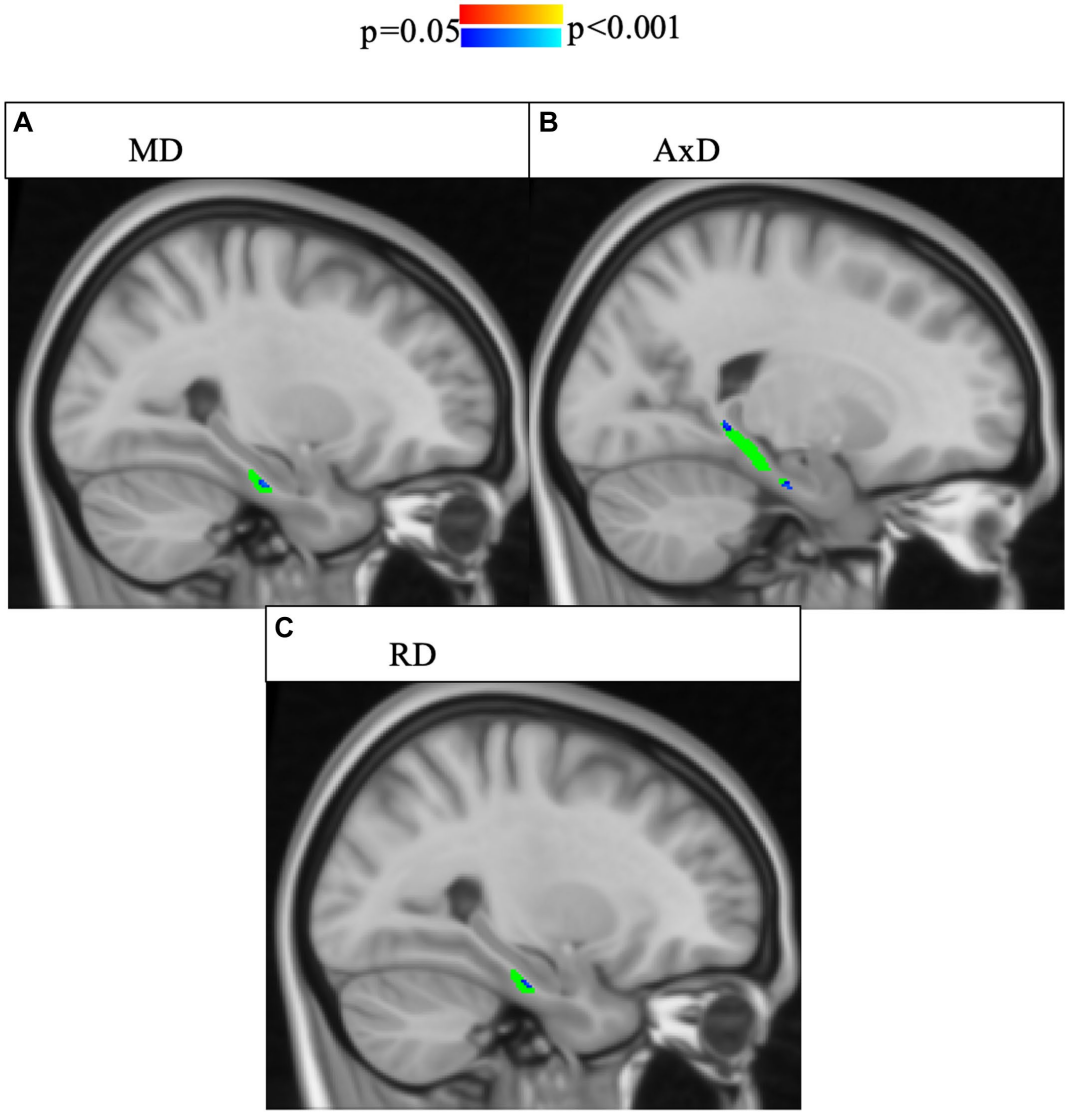
Interaction effects between cohort and age were confirmed by voxel-wise analysis with multiple comparisons correction of the whole right lower cingulum. Significant interactions (*p* < 0.05, corrected for multiple comparisons using threshold-free cluster enhancement) were found for areas of the right cingulum and are highlighted in blue shades. The region of interest (lower cingulum) is in green. Associations between increased age and increased **(A)** MD, **(B)** AxD, and **(C)** RD were stronger in SCD than in controls. FA is not pictured because no voxels showed a significant interaction effect.

As the risk for dementia increases with age (59), we were interested in the impact age had on the relationship between microstructure and story recall. To increase statistical power, our models only have three covariates each, particularly given the correlation between memory performance and group, as well as between memory performance and age. Model 2 includes age but does not include memory performance while Model 3 includes age but does not include group. In Model 2, there was a significant interaction effect between age and group in MD and AxD in right lower cingulum, which was robust to the removal of outliers (Figure 3). Voxel-wise analyses with multiple comparisons correction confirmed this finding, with the addition of significant interaction effects for RD in a subset of voxels (Figure 4). A significant interaction effect for MD and AxD was also observed in the left lower cingulum after the removal of outliers. However, when outliers were included the *p* value for MD increased from 0.005 to 0.078 and the p value for AxD increased from 0.003 to 0.025. All other findings were robust to the removal of outliers (Supplementary Table S5). In contrast, Model 3 did not show any significant interaction effects, indicating memory performance alone was not sensitive to the same group differences as SCD status despite the strong correlations between them (Supplementary Tables S6–S8). There were no significant interaction effects for cortical thickness in any of the regions studied (Supplementary Tables S8, S9). All findings were robust to the removal of outliers.

In post-hoc analyses, compared to controls, the SCD group showed a stronger correlation with age in MD, AxD, and RD, as well as a steeper slope, indicating an accelerated aging effect (Figure 3). There was a significant main effect with age in all imaging metrics studied. Across both groups, FA decreased with age (*ß* = −0.00216, *p* < 0.001), MD increased with age (*ß* = 2.51*10^−4^, *p* < 0.001), AxD increased with age (*ß* = 1.64*10^−6^, *p* < 0.001), and RD increased with age (*ß* = 3.06*10^−6^, *p* < 0.001). Left entorhinal (*ß* = −0.00654, *p* < 0.001), right entorhinal (*ß* = 8.00*10^−3^, *p* < 0.001), and right temporal pole (*ß* = −0.00504, *p* < 0.001) cortical thickness all decreased with age. All findings were robust to the removal of outliers.

### 3.4 Correlations between microstructure and cortical thickness

**Figure 5:**
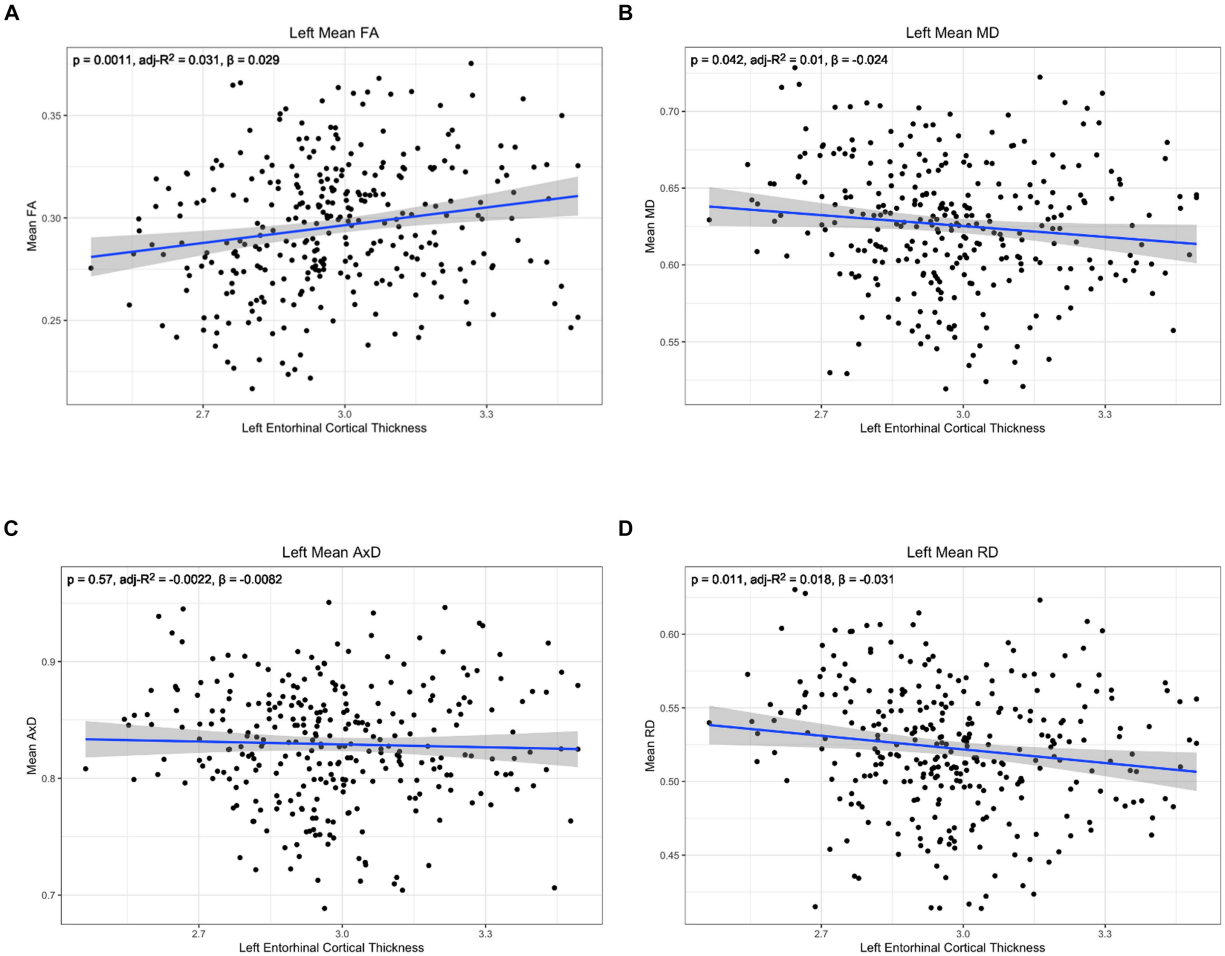
Correlation between diffusion metrics in the whole left lower cingulum and thickness of the left entorhinal cortex for (A) FA (B) MD (C) AxD and (D) RD. The correlation is significant for FA (A), MD (B), and RD (D). Diffusivity values are reported with units of 10^−3^ mm^2^/s.

**Figure 6:**
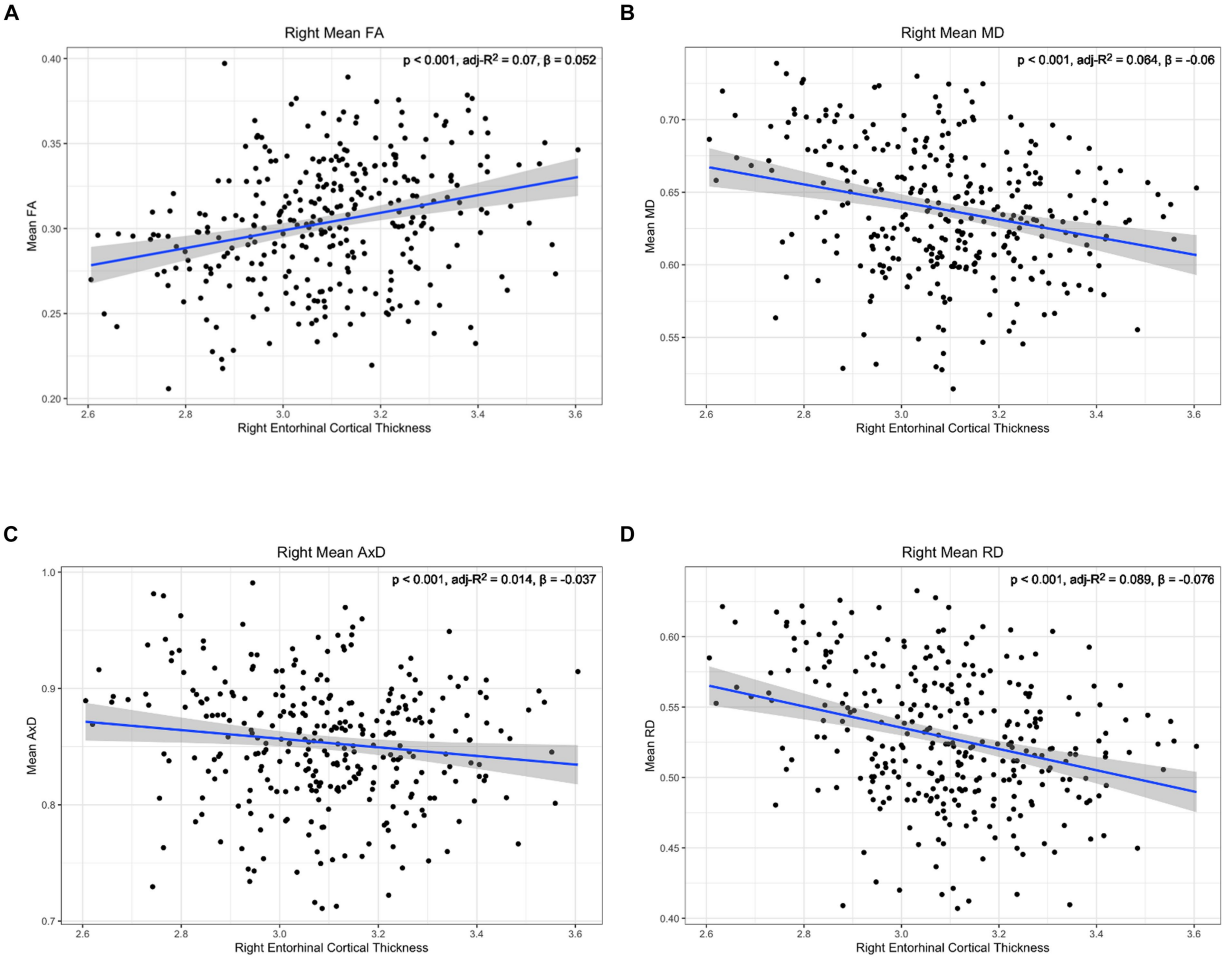
Correlation between diffusion metrics in the whole right lower cingulum and thickness of the right entorhinal cortex for (A) FA (B) MD (C) AxD and (D) RD. The correlation is significant for all diffusion metrics. Diffusivity values are reported with units of 10^−3^ mm^2^/s.

**Figure 7:**
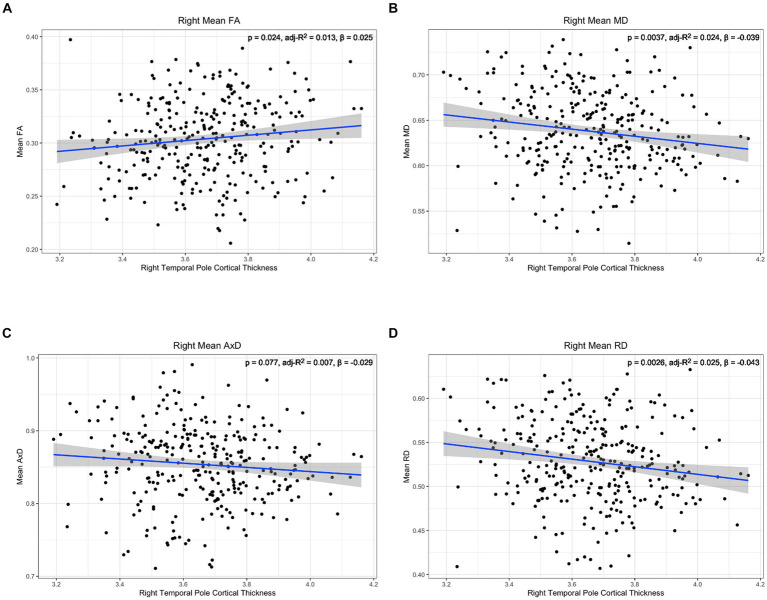
Correlation between diffusion metrics in the whole right lower cingulum and thickness of the right temporal pole for (A) FA (B) MD (C) AxD and (D) RD. The correlation is significant for FA (A), MD (B), and RD (D). Diffusivity values are reported with units of 10^−3^ mm^2^/s.

To further characterize the relationship between lower cingulum microstructure and atrophy, we modeled group differences in the relationship between each diffusion metric and cortical thickness of bilateral entorhinal cortex and right temporal pole using Model 4. The model was applied independently for the two hemispheres. There were no significant interaction effects between cortical thickness and group in any of the tests of Model 4 (Supplementary Tables S10–S12). However, there were significant main effects for diffusion microstructure across groups. Post-hoc analyses of diffusion microstructure by cortical thickness across groups revealed reduced cortical thicknesses in the left entorhinal, right entorhinal, and right temporal pole were all associated with decreased FA, increased MD, and increased RD (Figures 5–7). Additionally, reduced cortical thickness in right entorhinal cortex was associated with increased AxD (Figure 6). After adjusting for age, the associations between diffusion microstructure and cortical thickness were no longer significant (Supplementary Tables S13–S15).

## 4 Discussion

None of the cortical thickness measures showed any interaction between SCD status and age, SCD status and memory performance, or age and memory performance. Only diffusion metrics showed interaction effects between SCD status, memory, age, and neurobiological changes.

There was significant correlation between memory and all the diffusion markers examined (FA, MD, RD, and AxD) only in the SCD group. We found that decreased FA, increased MD, and increased RD had a stronger correlation with poor memory performance in SCD. This diffusion profile is typically associated with demyelination (60) or axonal loss (61, 62). However, both demyelination and axonal loss are associated with reduced AxD (63, 64). Here we see increased AxD, which has previously been observed in vasogenic edema associated with stroke (65), Monte Carlo simulations and tissue phantom models of vasogenic edema (66), and increased immunoreactivity associated with encephalitis (67, 68). Therefore, the diffusion profile noted here in SCD may indicate a mixed pathology of neurodegeneration and neuroinflammation. Our finding that cortical thickness and volume did not show any interaction between SCD status and memory performance further indicates that this diffusion profile is not explained by neurodegeneration alone. The effect sizes (adjusted *R*^2^) of the associations between memory and diffusion metrics are small, but SCD and controls show different biological correlates with memory performance.

When including age in our statistical modeling, participants with SCD present with an accelerated aging effect in diffusion microstructure, showing steeper variations with age. Similar to our findings in memory performance, this accelerated aging effect was noted for increased MD, RD, and AxD. As noted above, an increase in both RD and AxD is more suggestive of a mixed neurodegeneration and neuroinflammation pathology, rather than neurodegeneration alone (69–71). Cortical thickness did not capture this accelerated aging effect, further supporting that neurodegeneration alone was insufficient to explain these diffusion findings. The interaction between memory performance and age was not significant for any diffusion, cortical thickness, or cortical volume measures. Thus, SCD status was more sensitive than memory performance to differences in diffusion microstructure associated with aging.

Previous research investigating DTI in SCD has largely only included FA and MD in their analyses. Of these studies, some have reported no statistically significant findings in the lower cingulum (37, 72, 73). These mixed findings may reflect, in part, the presence of field inhomogeneities reducing the signal-to-noise ratio of the diffusion images in the lower cingulum (74), as many studies do report reduced FA and increased MD in SCD in bilateral cingulum (26, 29, 42) or increased MD in SCD in the left lower cingulum (75). These later data are comparable to our findings of increased MD and reduced FA in right lower cingulum with age and memory performance in SCD.

Studies that do include AxD and RD in their analyses also have mixed results, with some studies finding no changes in the lower cingulum (35, 36, 76). However, other studies found increased RD in bilateral lower cingulum (34, 77) and in left lower cingulum (78). While other studies have found increased AxD in SCD (79, 80), none of these have localized to the lower cingulum. In our results, we find increased RD and AxD in the right lower cingulum related to age and poor memory performance in SCD, with few significant results in the left. This contrasts with past studies that found increased RD and no changes in AxD in the left lower cingulum.

In mild cognitive impairment, increased RD and MD, but not AxD, were found to be associated with low cognitive performance (81, 82), representing a diffusion profile more suggestive of demyelination (83) and neurodegeneration (84). Prior research has shown a nonmonotonic trajectory of diffusion metrics in early AD, suggestive of an early inflammatory process followed by a neurodegenerative process (69–71). This includes an increase in free water, MD, and neurite density during the “inflammatory” stage followed by a decrease during the “degeneration” stage (71). Thus, our findings in SCD may be capturing an earlier, more inflammatory timepoint in the AD course occurring in parallel with neurodegeneration and the deposition of protein aggregates.

While we found group differences in cortical thickness in bilateral entorhinal cortex and right temporal pole, the correlations between cortical thickness and diffusion microstructural metrics did not differ between groups. Both AD and aging-related neurodegeneration are typically greater on the left than on the right (85), but here we found group differences in cortical thinning in more regions on the right than on the left. Cortical thinning in our study was associated with decreased FA, increased RD, and increased MD in the left lower cingulum and right temporal pole, a profile suggestive of demyelination (83) and suggestive of diffusion microstructural changes related to aging (86). In the right hemisphere, we found that cortical thinning was associated with increased AxD in addition to decreased FA, increased MD, and increased RD, suggesting a more mixed pathology, albeit with a small effect size.

In summary, DTI appeared to be more sensitive than cortical thickness to the associations between SCD, memory, and age. DTI metrics are aggregate measures of water diffusivity across microstructural compartments (87) and may reflect combinations of pathologies implicated in early AD, including demyelination, neurodegeneration, protein aggregation, neurovascular abnormalities, disrupted connectivity, and inflammation (88, 89). Thus, the combined effects of mixed pathology may increase the sensitivity of the DTI metrics to microstructural variations with age and cognition.

Strengths of this study include the large number of subjective cognitive decline participants scanned on the same MRI machine, at the same location, with the same protocol. This allowed us to achieve better sensitivity in the diffusion metric analysis. The participants have a diversity of years of education and income brackets. They are also evenly distributed between genders. No statistically significant differences in demographics were observed between cohorts.

Limitations of this study include a lack of ethnic diversity in the cohort. There also is no longitudinal data, Alzheimer’s biomarkers, or genetic data available from this dataset. Due to the lack of biomarker and longitudinal data in this study, it is difficult to interpret what pathologies underlie the changes observed in diffusion metrics here. Future work will apply similar analyses to a smaller dataset with better biomarker data and longitudinal follow up. Magnetization transfer imaging data available from Cam-CAN may further clarify the MRI microstructure variations in this particular cohort.

## Data availability statement

The original contributions presented in the study are included in the article/Supplementary material, further inquiries can be directed to the corresponding author.

## Ethics statement

The studies involving humans were approved by Cambridgeshire 2 Research Ethics Committee (reference: 10/H0308/50). The studies were conducted in accordance with the local legislation and institutional requirements. The participants provided their written informed consent to participate in this study. Written informed consent was obtained from the individual(s) for the publication of any potentially identifiable images or data included in this article.

## Author contributions

RF: Formal analysis, Methodology, Visualization, Writing – original draft, Writing – review & editing. YS: Methodology, Writing – original draft, Writing – review & editing. AM: Supervision, Writing – original draft, Writing – review & editing. RB: Writing – review & editing, Formal analysis. HR: Supervision, Writing – original draft, Writing – review & editing. ML: Funding acquisition, Project administration, Supervision, Writing – original draft, Writing – review & editing.
