# Supplementary material for: Diffusion imaging markers of accelerated aging of the lower cingulum in subjective cognitive decline
Source: Front Neurol. 2024 May 9;15:1360273. doi: 10.3389/fneur.2024.1360273 (PMC11111894; doi:10.3389/fneur.2024.1360273)
Supplement: Supplementary file 1 [file Table_1.docx]

**Supplemental Table 1: Mean ROI cortical thickness, in mm.** All nonsignificant regions analyzed. SD=standard deviation

|  | SCD (N=125 &) | Control (N=197 &) | Test statistic | p value | Cohen’s D |
| --- | --- | --- | --- | --- | --- |
| **Left Parahippocampal** |  |  | t=0.437 | 0.663 | 0.05 |
| Mean (SD) | 2.532 (0.220) | 2.521 (0.225) |  |  |  |
| Range | 2.007 - 3.143 | 1.965 - 3.028 |  |  |  |
| **Right Parahippocampal** |  |  | t=0.516 | 0.606 | 0.06 |
| Mean (SD) | 2.552 (0.144) | 2.543 (0.139) |  |  |  |
| Range | 2.205 - 2.926 | 2.165 - 2.909 |  |  |  |
| **Left Inferior Temporal** |  |  | t=0.271 | 0.787 | 0.03 |
| Mean (SD) | 2.787 (0.075) | 2.784 (0.082) |  |  |  |
| Range | 2.609 - 2.966 | 2.572 - 2.980 |  |  |  |
| **Right Inferior Temporal** |  |  | t=-0.410 | 0.682 | -0.05 |
| Mean (SD) | 2.883 (0.073) | 2.886 (0.073) |  |  |  |
| Range | 2.753 - 3.078 | 2.682 - 3.079 |  |  |  |
| **Left Middle Temporal** |  |  | t=-0.518 | 0.605 | -0.06 |
| Mean (SD) | 2.726 (0.056) | 2.730 (0.065) |  |  |  |
| Range | 2.587 - 2.859 | 2.555 - 2.896 |  |  |  |
| **Right Middle Temporal** |  |  | t=0.372 | 0.71 | 0.04 |
| Mean (SD) | 2.882 (0.061) | 2.879 (0.068) |  |  |  |
| Range | 2.736 - 3.046 | 2.704 - 3.042 |  |  |  |
| **Left Inferior Parietal** |  |  | t=0.236 | 0.814 | 0.03 |
| Mean (SD) | 2.375 (0.062) | 2.373 (0.056) |  |  |  |
| Range | 2.242 - 2.518 | 2.249 - 2.514 |  |  |  |
| **Right Inferior Parietal** |  |  | t=-0.122 | 0.903 | -0.01 |
| Mean (SD) | 2.493 (0.053) | 2.494 (0.047) |  |  |  |
| Range | 2.378 - 2.612 | 2.374 - 2.614 |  |  |  |
| **Left Superior Parietal** |  |  | t=0.583 | 0.561 | 0.07 |
| Mean (SD) | 2.020 (0.073) | 2.015 (0.065) |  |  |  |
| Range | 1.841 - 2.184 | 1.836 - 2.172 |  |  |  |
| **Right Superior Parietal** |  |  | t=0.815 | 0.416 | 0.10 |
| Mean (SD) | 2.104 (0.078) | 2.097 (0.070) |  |  |  |
| Range | 1.901 - 2.297 | 1.909 - 2.290 |  |  |  |
| **Left Precuneus** |  |  | t=0.686 | 0.494 | 0.08 |
| Mean (SD) | 2.280 (0.076) | 2.274 (0.064) |  |  |  |
| Range | 2.087 - 2.470 | 2.083 - 2.426 |  |  |  |
| **Right Precuneus** |  |  | t=1.59 | 0.114 | 0.18 |
| Mean (SD) | 2.338 (0.064) | 2.327 (0.062) |  |  |  |
| Range | 2.176 - 2.457 | 2.168 - 2.497 |  |  |  |
| **Left Posterior Cingulate** |  |  | t=-0.480 | 0.631 | -0.06 |
| Mean (SD) | 2.366 (0.086) | 2.371 (0.077) |  |  |  |
| Range | 2.154 - 2.558 | 2.167 - 2.558 |  |  |  |
| **Right Posterior Cingulate** |  |  | t=0.707 | 0.480 | 0.08 |
| Mean (SD) | 2.358 (0.101) | 2.350 (0.092) |  |  |  |
| Range | 2.135 - 2.559 | 2.094 - 2.579 |  |  |  |

**Supplemental Table 2: Mean ROI Volume, in mm^3^.** All nonsignificant regions analyzed. SD=standard deviation.

|  | SCD (N=125 &) | Control (N=197 &) | Test statistic | p value | Cohen’s D |
| --- | --- | --- | --- | --- | --- |
| **Left Hippocampus** |  |  | t=-1.07 | 0.288 | -0.12 |
| Mean (SD) | 2.439 (0.238) | 2.469 (0.247) |  |  |  |
| Range | 1.887 - 3.042 | 1.816 - 3.029 |  |  |  |
| **Right Hippocampus** |  |  | t=-0.366 | 0.715 | -0.04 |
| Mean (SD) | 2.405 (0.241) | 2.415 (0.241) |  |  |  |
| Range | 1.781 - 2.990 | 1.825 - 2.979 |  |  |  |
| **Left Lower Cingulum** |  |  | t=0.483 | 0.630 | 0.06 |
| Mean (SD) | 1.176 (0.129) | 1.168 (0.129) |  |  |  |
| Range | 0.907 - 1.514 | 0.819 - 1.530 |  |  |  |
| **Right Lower Cingulum** |  |  | t=-0.615 | 0.539 | -0.07 |
| Mean (SD) | 1.074 (0.137) | 1.084 (0.145) |  |  |  |
| Range | 0.695 - 1.442 | 0.697 - 1.430 |  |  |  |

& There were 327 participants over the age of 55 with anatomical T1w images available. Two control participants and three SCD participants were excluded from the cortical thickness and brain structure volume analyses due to segmentation errors, for a total of 322 segmentations as detailed in these tables.

**Supplemental Table 3: Left Lower Cingulum regional diffusion metric mean values by delayed story recall and group (Model 1).** No statistically significant interaction effects or main effects were found. Diffusivity values are reported with units of 10^-3^ mm^2^/s. SE=Standard error.

|  | Left FA | Left MD | Left AxD | Left RD |
| --- | --- | --- | --- | --- |
| **Delayed Story Recall β** | -4.37e-04 | 4.41e-04 | 1.16e-04 | 3.41e-04 |
|  | SE = 6.03e-04 | SE = 8.24e-04 | SE = 1.01e-03 | SE = 8.39e-04 |
|  | t = -0.726 | t = 0.535 | t = 0.114 | t = 0.406 |
|  | p = 0.469 | p = 0.593 | p = 0.909 | p = 0.685 |
|  |  |  |  |  |
| **Group (SCD vs Control) β** | -5.60e-04 | -6.95e-03 | -5.88e-03 | -6.36e-03 |
|  | SE = 3.73e-03 | SE = 4.89e-03 | SE = 6.02e-03 | SE = 5.07e-03 |
|  | t = -0.150 | t = -1.420 | t = -0.976 | t = -1.255 |
|  | p = 0.881 | p = 0.157 | p = 0.330 | p = 0.210 |
|  |  |  |  |  |
| **Story x Group β** | 9.46e-04 | -2.13e-03 | -1.91e-03 | -2.04e-03 |
|  | SE = 8.58e-04 | SE = 1.15e-03 | SE = 1.41e-03 | SE = 1.18e-03 |
|  | t = 1.102 | t = -1.859 | t = -1.356 | t = -1.724 |
|  | p = 0.271 | p = 0.064 | p = 0.176 | p = 0.086 |
|  |  |  |  |  |
| **Adjusted R^2^** | -0.005 | 0.010 | 0.003 | 0.007 |

**Supplemental Table 4: Cortical Thickness by delayed story recall and group, in mm (Model 1).** No statistically significant interaction effects or main effects were found. SE=Standard error.

|  | Left Entorhinal | Right Entorhinal | Right Temporal Pole |
| --- | --- | --- | --- |
| **Delayed Story Recall β** | 4.91e-03 | 2.69e-04 | -1.28e-03 |
|  | SE = 3.81e-03 | SE = 3.54e-03 | SE = 3.48e-03 |
|  | t = 1.288 | t = 0.076 | t = -0.367 |
|  | p = 0.199 | p = 0.940 | p = 0.714 |
| **Group (SCD vs Control) β** | -5.30e-02 | -5.61e-02 | -4.49e-02 |
|  | SE = 2.35e-02 | SE = 2.18e-02 | SE = 2.15e-02 |
|  | t = -2.252 | t = -2.579 | t = -2.088 |
|  | p = 0.025 | p = 0.010 | p = 0.038 |
| **Story x Group β** | -5.31e-03 | 2.29e-03 | 6.16e-03 |
|  | SE = 5.42e-03 | SE = 5.02e-03 | SE = 4.94e-03 |
|  | t = -0.979 | t = 0.455 | t = 1.248 |
|  | p = 0.328 | p = 0.649 | p = 0.213 |
| **Adjusted R^2^** | 0.014 | 0.016 | 0.014 |

**Supplemental Table 5: Left Lower Cingulum regional diffusion metric mean values by age and group (Model 2).** Statistically significant interaction effects were found for MD and AxD, but these were not robust to the inclusion of outliers (values including outliers are noted in parentheses where different from values without outliers). A main effect of age was found for FA, MD, and RD. When outliers were included, a main effect of age was also found for AxD. Diffusivity values are reported with units of 10^-3^ mm^2^/s. SE=Standard error.

|  | Left FA | Left MD | Left AxD | Left RD |
| --- | --- | --- | --- | --- |
| **Age β** | -1.58e-03 | 1.43e-03 | 5.56e-04 | 2.02e-03 |
|  | SE = 2.34e-04 | SE = 3.05e-04 | SE = 3.92e-04 | SE = 3.10e-04 |
|  | t = -6.767 | t = 4.685 | t = 1.416 | t = 6.509 |
|  | ***p < 0.001*** | ***p < 0.001*** | ***p = 0.158 (0.012)*** | ***p < 0.001*** |
| **Group (SCD vs Control) β** | 7.90e-04 | -7.50e-03 | -6.10e-03 | -7.22e-03 |
|  | SE = 3.35e-03 | SE = 4.35e-03 | SE = 5.63e-03 | SE = 4.41e-03 |
|  | t = 0.236 | t = -1.723 | t = -1.083 | t = -1.636 |
|  | p = 0.814 | p = 0.086 (0.216) | p = 0.280 | p = 0.103 |
| **Age x Group β** | 2.84e-04 | 1.37e-03 | 2.28e-03 | 7.62e-04 |
|  | SE = 3.74e-04 | SE = 4.89e-04 | SE = 6.29e-04 | SE = 4.94e-04 |
|  | t = 0.760 | t = 2.791 | t = 3.630 | t = 1.542 |
|  | p = 0.448 | ***p = 0.006 (0.079)*** | ***p < 0.001 (0.025)*** | p = 0.124 |
| **Adjusted R^2^** | 0.164 | 0.191 | 0.096 | 0.225 |

**Supplemental Table 6: Right Lower Cingulum regional diffusion metric mean values by age and delayed story recall (Model 3).** No statistically significant interaction effects or main delayed story recall effects were found. A main effect of age was found (refer to Figure 3 in the main text). Diffusivity values are reported with units of 10^-3^ mm^2^/s. SE=Standard error.

|  | Right FA | Right MD | Right AxD | Right RD |
| --- | --- | --- | --- | --- |
| **Delayed Story Recall β** | -1.53e-04 | -7.03e-04 | -1.18e-03 | -7.31e-04 |
|  | SE = 4.28e-04 | SE = 4.93e-04 | SE = 6.73e-04 | SE = 4.95e-04 |
|  | t = -0.357 | t = -1.426 | t = -1.756 | t = -1.478 |
|  | p = 0.721 | p = 0.155 | p = 0.080 | p = 0.141 |
| **Age β** | -2.15e-03 | 2.83e-03 | 2.08e-03 | 3.29e-03 |
|  | SE = 2.00e-04 | SE = 2.29e-04 | SE = 3.13e-04 | SE = 2.31e-04 |
|  | t = -10.732 | t = 12.365 | t = 6.646 | t = 14.228 |
|  | ***p < 0.001*** | ***p < 0.001*** | ***p < 0.001*** | ***p < 0.001*** |
| **Age x Story β** | -1.72e-05 | -8.37e-05 | -1.10e-04 | -4.22e-05 |
|  | SE = 4.80e-05 | SE = 5.53e-05 | SE = 7.54e-05 | SE = 5.55e-05 |
|  | t = -0.358 | t = -1.515 | t = -1.454 | t = -0.760 |
|  | p = 0.721 | p = 0.131 | p = 0.147 | p = 0.448 |
| **Adjusted R^2^** | 0.265 | 0.351 | 0.149 | 0.410 |

**Supplemental Table 7: Left Lower Cingulum regional diffusion metric mean values by age and delayed story recall (Model 3).** No statistically significant interaction effects or main delayed story recall effects were found. A main effect of age was found (refer to Supplemental Table 5). SE=Standard error.

|  | Left FA | Left MD | Left AxD | Left RD |
| --- | --- | --- | --- | --- |
| **Delayed Story Recall β** | -6.62e-04 | 3.79e-04 | -2.47e-05 | 5.78e-04 |
|  | SE = 3.97e-04 | SE = 5.33e-04 | SE = 6.92e-04 | SE = 5.36e-04 |
|  | t = -1.669 | t = 0.711 | t = -0.036 | t = 1.078 |
|  | p = 0.096 | p = 0.478 | p = 0.972 | p = 0.282 |
| **Age β** | -1.54e-03 | 1.98e-03 | 1.43e-03 | 2.35e-03 |
|  | SE = 1.86e-04 | SE = 2.48e-04 | SE = 3.20e-04 | SE = 2.49e-04 |
|  | t = -8.287 | t = 7.993 | t = 4.466 | t = 9.463 |
|  | ***p < 0.001*** | ***p < 0.001*** | ***p < 0.001*** | ***p < 0.001*** |
| **Age x Story β** | -4.09e-05 | -2.17e-05 | -8.16e-05 | -2.41e-05 |
|  | SE = 4.45e-05 | SE = 6.04e-05 | SE = 7.82e-05 | SE = 5.97e-05 |
|  | t = -0.920 | t = -0.359 | t = -1.044 | t = -0.404 |
|  | p = 0.358 | p = 0.720 | p = 0.297 | p = 0.687 |
| **Adjusted R^2^** | 0.173 | 0.166 | 0.058 | 0.216 |

**Supplemental Table 8: Cortical Thickness by age and delayed story recall (Model 3):** No statistically significant interaction effects or main delayed story recall effects were found. A main effect of age was found (refer to main text). SE=Standard error.

|  | Left Entorhinal | Right Entorhinal | Right Temporal Pole |
| --- | --- | --- | --- |
| **Delayed Story Recall β** | -3.27e-04 | -1.08e-03 | -1.08e-03 |
|  | SE = 2.65e-03 | SE = 2.38e-03 | SE = 2.38e-03 |
|  | t = -0.124 | t = -0.452 | t = -0.452 |
|  | p = 0.902 | p = 0.652 | p = 0.652 |
| **Age β** | -7.30e-03 | -8.27e-03 | -8.27e-03 |
|  | SE = 1.25e-03 | SE = 1.11e-03 | SE = 1.11e-03 |
|  | t = -5.849 | t = -7.455 | t = -7.455 |
|  | ***p < 0.001*** | ***p < 0.001*** | ***p < 0.001*** |
| **Age x Story β** | 4.82e-05 | -3.14e-04 | -3.14e-04 |
|  | SE = 2.97e-04 | SE = 2.66e-04 | SE = 2.66e-04 |
|  | t = 0.162 | t = -1.180 | t = -1.180 |
|  | p = 0.871 | p = 0.239 | p = 0.239 |
| **Adjusted R^2^** | 0.094 | 0.150 | 0.150 |

**Supplemental Table 9: Cortical Thickness by age and group (Model 2):** No statistically significant interaction effects were found. Main effects of age and group were found (refer to Supplemental Table 8 and main text).

|  | Left Entorhinal | Right Entorhinal | Right Temporal Pole |
| --- | --- | --- | --- |
| **Age β** | -6.54e-03 | -8.00e-03 | -5.04e-03 |
|  | SE = 1.55e-03 | SE = 1.37e-03 | SE = 1.41e-03 |
|  | t = -4.212 | t = -5.825 | t = -3.577 |
|  | ***p < 0.001*** | ***p < 0.001*** | ***p < 0.001*** |
| **Group (SCD vs Control) β** | -4.67e-02 | -4.98e-02 | -4.24e-02 |
|  | SE = 2.21e-02 | SE = 1.98e-02 | SE = 2.04e-02 |
|  | t = -2.116 | t = -2.517 | t = -2.083 |
|  | ***p = 0.035*** | ***p = 0.012*** | ***p = 0.038*** |
| **age x Group β** | -1.40e-03 | -1.28e-06 | -1.70e-03 |
|  | SE = 2.47e-03 | SE = 2.20e-03 | SE = 2.26e-03 |
|  | t = -0.566 | t = -0.001 | t = -0.750 |
|  | p = 0.572 | p = 1.000 | p = 0.454 |
| **Adjusted R^2^** | 0.108 | 0.162 | 0.087 |

**Supplemental Table 10: Left Lower Cingulum regional diffusion metrics by left entorhinal cortex thickness and group (Model 4):** No statistically significant interaction effects or main effects of group were found. A main effect of thickness was found for FA (refer to Figure 5 of main text). Diffusivity values are reported with units of 10^-3^ mm^2^/s. SE=Standard error.

|  | Left FA | Left MD | Left AxD | Left RD |
| --- | --- | --- | --- | --- |
| **Thickness β** | 2.59e-02 | -2.15e-02 | -9.57e-03 | -2.75e-02 |
|  | SE = 1.10e-02 | SE = 1.46e-02 | SE = 1.80e-02 | SE = 1.51e-02 |
|  | t = 2.365 | t = -1.474 | t = -0.532 | t = -1.818 |
|  | ***p = 0.019*** | p = 0.141 | p = 0.595 | p = 0.070 |
| **Group β** | -2.87e-02 | 3.01e-02 | -5.43e-03 | 4.07e-02 |
|  | SE = 5.55e-02 | SE = 7.24e-02 | SE = 8.95e-02 | SE = 7.53e-02 |
|  | t = -0.517 | t = 0.416 | t = -0.061 | t = 0.541 |
|  | p = 0.606 | p = 0.678 | p = 0.952 | p = 0.589 |
| **Thickness x Group β** | 1.02e-02 | -1.24e-02 | 6.20e-04 | -1.61e-02 |
|  | SE = 1.87e-02 | SE = 2.43e-02 | SE = 3.01e-02 | SE = 2.53e-02 |
|  | t = 0.545 | t = -0.511 | t = 0.021 | t = -0.634 |
|  | p = 0.586 | p = 0.610 | p = 0.984 | p = 0.527 |
| **Adjusted R^2^** | 0.026 | 0.011 | -0.008 | 0.019 |

**Supplemental Table 11: Right Lower Cingulum regional diffusion metrics by right entorhinal cortex thickness and group (Model 4):** No statistically significant interaction effects or main effects of group were found. A main effect of thickness was found for FA, MD, and RD (refer to Figure 6 of the main text). Diffusivity values are reported with units of 10^-3^ mm^2^/s. SE=Standard error.

|  | Right FA | Right MD | Right AxD | Right RD |
| --- | --- | --- | --- | --- |
| **Thickness β** | 4.24e-02 | -5.14e-02 | -3.39e-02 | -6.23e-02 |
|  | SE = 1.32e-02 | SE = 1.61e-02 | SE = 1.99e-02 | SE = 1.68e-02 |
|  | t = 3.227 | t = -3.187 | t = -1.705 | t = -3.700 |
|  | ***p = 0.001*** | ***p = 0.002*** | p = 0.089 | ***p < 0.001*** |
| **Group β** | -7.42e-02 | 8.25e-02 | 4.13e-02 | 1.12e-01 |
|  | SE = 6.82e-02 | SE = 8.42e-02 | SE = 1.04e-01 | SE = 8.78e-02 |
|  | t = -1.087 | t = 0.980 | t = 0.398 | t = 1.277 |
|  | p = 0.278 | p = 0.328 | p = 0.691 | p = 0.202 |
| **Thickness x Group β** | 2.30e-02 | -2.72e-02 | -1.51e-02 | -3.59e-02 |
|  | SE = 2.21e-02 | SE = 2.73e-02 | SE = 3.36e-02 | SE = 2.85e-02 |
|  | t = 1.039 | t = -0.995 | t = -0.448 | t = -1.259 |
|  | p = 0.300 | p = 0.320 | p = 0.654 | p = 0.209 |
| **Adjusted R^2^** | 0.069 | 0.061 | 0.011 | 0.088 |

**Supplemental Table 12: Right Lower Cingulum regional diffusion metrics by right temporal pole cortex thickness and group (Model 4):** No statistically significant interaction effects or main effects of group were found. A main effect of thickness was found for FA, MD, and RD (refer to Figure 7 of the main text). Diffusivity values are reported with units of 10^-3^ mm^2^/s. SE=Standard error.

|  | Right FA | Right MD | Right AxD | Right RD |
| --- | --- | --- | --- | --- |
| **Thickness β** | 3.17e-02 | -4.82e-02 | -3.64e-02 | -5.06e-02 |
|  | SE = 1.50e-02 | SE = 1.81e-02 | SE = 2.20e-02 | SE = 1.93e-02 |
|  | t = 2.110 | t = -2.658 | t = -1.654 | t = -2.628 |
|  | ***p = 0.036*** | ***p = 0.008*** | p = 0.099 | ***p = 0.009*** |
| **Group β** | 6.25e-02 | -7.52e-02 | -5.31e-02 | -6.95e-02 |
|  | SE = 8.19e-02 | SE = 9.97e-02 | SE = 1.21e-01 | SE = 1.06e-01 |
|  | t = 0.763 | t = -0.754 | t = -0.439 | t = -0.656 |
|  | p = 0.446 | p = 0.451 | p = 0.661 | p = 0.512 |
| **Thickness x Group β** | -1.85e-02 | 2.06e-02 | 1.32e-02 | 2.01e-02 |
|  | SE = 2.24e-02 | SE = 2.73e-02 | SE = 3.30e-02 | SE = 2.90e-02 |
|  | t = -0.827 | t = 0.757 | t = 0.401 | t = 0.692 |
|  | p = 0.409 | p = 0.450 | p = 0.689 | p = 0.489 |
| **Adjusted R^2^** | 0.014 | 0.019 | 0.003 | 0.022 |

**Supplemental Table 13: Left Lower Cingulum regional diffusion metrics by left entorhinal cortex thickness, adjusted for age:** Cortical thickness by diffusion microstructure is no longer significant after adjusting for age (refer to Figure 5 in main text). Diffusivity values are reported with units of 10^-3^ mm^2^/s. SE=Standard error.

|  | Left FA | Left MD | Left AxD | Left RD |
| --- | --- | --- | --- | --- |
| **Thickness β** | 9.82e-03 | 4.49e-03 | 1.45e-02 | 1.98e-03 |
|  | SE = 8.58e-03 | SE = 1.12e-02 | SE = 1.46e-02 | SE = 1.14e-02 |
|  | t = 1.145 | t = 0.401 | t = 0.994 | t = 0.174 |
|  | p = 0.253 | p = 0.689 | p = 0.321 | p = 0.862 |
| **Age β** | -1.37e-03 | 1.98e-03 | 1.57e-03 | 2.30e-03 |
|  | SE = 1.93e-04 | SE = 2.57e-04 | SE = 3.31e-04 | SE = 2.58e-04 |
|  | t = -7.065 | t = 7.707 | t = 4.755 | t = 8.901 |
|  | ***p < 0.001*** | ***p < 0.001*** | ***p < 0.001*** | ***p < 0.001*** |
| **Adjusted R^2^** | 0.162 | 0.170 | 0.065 | 0.216 |

**Supplemental Table 14: Right Lower Cingulum regional diffusion metrics by right entorhinal cortex thickness, adjusted for age:** Cortical thickness by diffusion microstructure is no longer significant after adjusting for age (refer to Figure 6 in main text). Diffusivity values are reported with units of 10^-3^ mm^2^/s. SE=Standard error.

|  | Right FA | Right MD | Right AxD | Right RD |
| --- | --- | --- | --- | --- |
| **Thickness β** | 1.53e-02 | -7.45e-03 | 5.73e-03 | -1.49e-02 |
|  | SE = 1.01e-02 | SE = 1.17e-02 | SE = 1.60e-02 | SE = 1.18e-02 |
|  | t = 1.522 | t = -0.638 | t = 0.357 | t = -1.267 |
|  | p = 0.129 | p = 0.524 | p = 0.721 | p = 0.206 |
| **Age β** | -1.95e-03 | 2.82e-03 | 2.27e-03 | 3.20e-03 |
|  | SE = 2.10e-04 | SE = 2.44e-04 | SE = 3.36e-04 | SE = 2.44e-04 |
|  | t = -9.260 | t = 11.554 | t = 6.768 | t = 13.083 |
|  | ***p < 0.001*** | ***p < 0.001*** | ***p < 0.001*** | ***p < 0.001*** |
| **Adjusted R^2^** | 0.266 | 0.344 | 0.139 | 0.408 |

**Supplemental Table 15: Right Lower Cingulum regional diffusion metrics by right temporal pole cortex thickness, adjusted for age:** Cortical thickness by diffusion microstructure is no longer significant after adjusting for age (refer to Figure 7 in main text). Diffusivity values are reported with units of 10^-3^ mm^2^/s. SE=Standard error.

|  | Right FA | Right MD | Right AxD | Right RD |
| --- | --- | --- | --- | --- |
| **Thickness β** | -4.09e-03 | 1.77e-03 | 2.42e-03 | 4.47e-03 |
|  | SE = 9.99e-03 | SE = 1.15e-02 | SE = 1.58e-02 | SE = 1.17e-02 |
|  | t = -0.410 | t = 0.154 | t = 0.153 | t = 0.383 |
|  | p = 0.682 | p = 0.878 | p = 0.878 | p = 0.702 |
| **Age β** | -2.09e-03 | 2.86e-03 | 2.20e-03 | 3.32e-03 |
|  | SE = 2.04e-04 | SE = 2.36e-04 | SE = 3.24e-04 | SE = 2.36e-04 |
|  | t = -10.237 | t = 12.153 | t = 6.809 | t = 14.053 |
|  | ***p < 0.001*** | ***p < 0.001*** | ***p < 0.001*** | ***p < 0.001*** |
| **Adjusted R^2^** | 0.259 | 0.340 | 0.135 | 0.402 |
